# COVID-19-Associated Mucormycosis: Identifying Mortality Predictors in a Retrospective Cohort Study

**DOI:** 10.1093/ofid/ofaf674

**Published:** 2025-11-03

**Authors:** Zahra Ghavami, Mahboubeh Haddad, Fereshte Sheybani, Matin Shirazinia, Maliheh Dadgar Moghadam

**Affiliations:** Department of Infectious Diseases and Tropical Medicine, Faculty of Medicine, Mashhad University of Medical Sciences, Mashhad, Iran; Department of Infectious Diseases and Tropical Medicine, Faculty of Medicine, Mashhad University of Medical Sciences, Mashhad, Iran; Department of Infectious Diseases and Tropical Medicine, Faculty of Medicine, Mashhad University of Medical Sciences, Mashhad, Iran; Department of Infectious Diseases and Tropical Medicine, Faculty of Medicine, Mashhad University of Medical Sciences, Mashhad, Iran; Department of Community Medicine, Faculty of Medicine, Mashhad University of Medical Sciences, Mashhad, Iran

**Keywords:** corticosteroids, COVID-19 associated mucormycosis, diabetes mellitus, mortality, rhino-orbital-cerebral mucormycosis

## Abstract

**Background:**

COVID-19-associated mucormycosis (CAM) has emerged as a serious complication of COVID-19, exacerbated by the use of immunosuppressive treatments. This study aims to assess the clinical outcomes and identify predictors of mortality in adult patients with CAM.

**Methods:**

This retrospective cohort study was conducted at a major referral center in Mashhad, Iran, from the start of the COVID-19 pandemic until the end of its fifth wave in Iran.

**Results:**

Of the 81 patients studied, 55.6% were male, with a median age of 60.0 years. Diabetes mellitus was the most common comorbidity, present in 76.5% of patients. Corticosteroids were used in 90.1% of cases. One-month survival was 61.7%, decreasing to 51.9% at three months and stabilizing at 50.6% at six and twelve months. CRP levels were associated with increased mortality (*P*-value = .001), while longer antifungal treatment duration was linked to reduced mortality (*P*-value < .001). Moreover, palatal necrosis (HR: 2.12) and the use of posaconazole (HR: 0.16) were significantly associated with the risk of mortality. While the former was a risk factor, the latter reduced the mortality rate.

**Conclusions:**

Our study contributes to the growing body of evidence on the clinical and epidemiological characteristics of CAM, highlighting the need for early diagnosis, risk factor identification, and prompt, prolonged treatment to enhance patient outcomes. Elevated CRP was identified as a predictor of mortality in CAM. Future research should aim to refine risk stratification methods and optimize therapeutic protocols to address the high mortality rates associated with this severe condition.

The COVID-19 pandemic (an acute respiratory infectious disease), caused by SARS-CoV-2 (an enveloped, positive-sense RNA coronavirus responsible for causing COVID-19), continues to affect populations worldwide. While the disease manifests in a wide range of clinical symptoms, from mild to life-threatening, it is particularly concerning for the association with severe complications, such as pneumonia and secondary bacterial or fungal infections [1]. The identification and management of these complications pose significant challenges in clinical practice. One such life-threatening complication is mucormycosis, a rare but aggressive fungal infection [2]. This opportunistic infection primarily affects immunocompromised individuals, particularly those with uncontrolled diabetes or hematologic malignancies [3].

Patients with COVID-19, especially those with severe cases, often experience lymphopenia and immune suppression, which, along with the use of anti-inflammatory treatments such as corticosteroids, increases their susceptibility to invasive fungal infections like mucormycosis [4]. Mucormycosis is a highly fatal fungal infection that has emerged as a significant complication in patients with COVID-19 [4]. If not diagnosed and treated promptly, it can lead to poor outcomes due to its rapid and invasive nature [3].

During the COVID-19 pandemic, there has been an alarming rise in reported cases of mucormycosis among patients with SARS-CoV-2 infection [5, 6]. These cases tend to develop during or after the recovery phase of COVID-19, especially in those treated with corticosteroids, a common therapy for severe COVID-19 cases [7]. This study aims to identify clinical predictors of mortality and assess the clinical outcomes of COVID-19-associated mucormycosis (CAM) in patients hospitalized in a major referral center in Mashhad, Iran.

## METHODS

This retrospective cohort study was conducted to evaluate the clinical outcomes of adult patients diagnosed with mucormycosis following or during COVID-19 infection. The study population included patients hospitalized at Imam Reza Teaching Hospital, the largest hospital in northeastern Iran (a 1000-bed tertiary referral center), which serves as a major center for both COVID-19 and invasive mold infections.

The study was performed from the first wave to the fifth wave of COVID-19 in Iran, covering the period from 1 March 2020, to 18 December 2021.

### Study Population and Data Collection

The study included all patients admitted with a confirmed diagnosis of CAM. The inclusion criteria were as follows: (1) age ≥18 years, (2) confirmed diagnosis of COVID-19 by RT-PCR, and (3) confirmed mucormycosis diagnosis based on histopathological evidence (identifying broad, non-septate hyphae with right-angle branching that infiltrate blood vessels) [8] of fungal invasion with/without positive culture for Mucorales. Exclusion criteria included patients with incomplete medical records regarding diagnostic confirmation or those with alternative fungal diagnoses.

Researchers collected data from hospital records, including demographic information, underlying conditions, clinical findings, the time interval from the diagnosis of COVID-19 to the appearance of mucormycosis symptoms, laboratory findings (only the initial record), histopathological findings, imaging results, as well as treatment regimen, treatment duration, and clinical outcomes at discharge.

At the end of the study, a follow-up was conducted to evaluate the survival status of all patients, with survival status recorded as either “alive” or “deceased.”

### Statistical Analysis

The normality of continuous variables was assessed using the Shapiro–Wilk test. Descriptive statistics were used to summarize the data, including frequency, percentage, and distribution for categorical variables, and median (percentile 25 to percentile 75) for continuous variables. STATA version 14 was used for statistical analysis. Survival analysis was conducted using log-rank tests and semi-parametric Cox regression. Additionally, Kaplan–Meier curves were used to illustrate patient survival.

Given that the number of outcome-positive cases in the analysis was relatively low, we presented only the unadjusted model and the age-adjusted model, as age is a known risk factor for mortality in patients with mucormycosis [9, 10]. The proportional hazards assumption was tested using statistical tests and visual inspection of Schoenfeld residuals. Furthermore, collinearity in multivariable models was assessed using the *collin* option in STATA. All plots were generated using RStudio.

### Ethics

Informed consent was obtained from all patients or their legally authorized representatives. The study was approved by the ethics committee of Mashhad University of Medical Sciences under code IR.MUMS.MEDICAL.REC.1400.805.

## RESULTS

### Patient Characteristics

Among the 81 patients with CAM, the majority were male (45 patients, 55.6%), with a median age of 60.0 years (interquartile range [IQR], 54.0–67.0). A total of 25 patients (30.9%) were aged 65 years or older. Diabetes mellitus was the most common underlying condition, observed in 62 patients (76.5%), of whom 9 (14.5%) were newly diagnosed at admission. Of the 45 diabetic patients with available laboratory data on diabetic ketoacidosis status, 3 (6.7%) had diabetic ketoacidosis. Chemotherapy was administered to 6 patients (7.4%), and immunodeficiency conditions were observed in 10 patients (12.4%). Hematologic malignancies or hematologic diseases were reported in 5 patients (6.2%), including acute lymphoblastic leukemia, acute myeloid leukemia (AML-M2), AML with myelodysplastic syndrome, non-Hodgkin lymphoma with prior splenectomy, and aplastic anemia, each diagnosed in 1 patient (1.2%). Solid tumors were noted in 2 patients (2.5%), specifically breast cancer and rhabdomyosarcoma. Organ transplantation was documented in 2 patients (2.5%), with one case of kidney transplantation and one case of liver transplantation. The median duration between COVID-19 infection and the onset of CAM was 15.0 days (IQR, 10.0–21.0) (Table 1).

**Table 1.** Characteristics of Patients With COVID-19-associated Mucormycosis

Variables	Possible (n = 81)
Age (y), median (percentile 25 to percentile 75)	60.0 (54.0 to 67.0)
Elderly (> 65 y), n (%)	25 (30.9)
Gender (male), n (%)	45 (55.6)
Underlying comorbidities, n (%)	
Immunocompromised (other than due to diabetes mellitus)	10 (12.4)
Diabetes mellitus	62 (76.5)
History of solid organ transplantation	2 (2.5)
Hematologic malignancy/disease	5 (6.2)
Other cancers	2 (2.5)
ESR/CKD	3 (3.7)
HIV/AIDS	1 (1.2)
Clinical manifestations, n (%)	
Headache	55 (67.9)
Fever	23 (28.4)
Facial pain	50 (61.7)
Facial numbness	46 (56.8)
Nasal discharge	28 (34.6)
Visual impairment	45 (55.6)
Proptosis	48 (59.3)
Periorbital swelling	49 (60.5)
Palatal necrosis	34 (42.0)
Nasal ulcer	35 (43.2)
Epistaxis	19 (23.5)
Facial paralysis	26 (32.1)
Facial swelling	57 (70.4)
Ptosis	54 (66.7)
Facial skin involvement	2 (2.5)
Symptom duration (d), median (percentile 25 to percentile 75)	4.0 (1.0 to 8.0)
Imaging findings, n (%)	
Orbital involvement (based on CT scan)	59 (72.8)
Brain involvement (based on CT scan)	26/55 (47.3)
Brain involvement (based on MRI)	23/38 (60.5)
Laboratory features, median (percentile 25 to percentile 75)	
ESR (mm/h)	80.0 (40.0 to 101.0), n = 67
CRP (mg/L)	99.0 (31.9 to 173.0), n = 74
Leukocytes (×10^3^/per µl)	11.5 (8.0 to 15.0)
Hemoglobin (mg/dL)	12.2 (10.1 to 13.7)
Platelets (×10^3^/per µl)	206.0 (139.0 to 287.0)
Blood sugar (mg/dL)	203.5 (144.0 to 289.0), n = 78
Amphotericin B formulation, n (%)	
Conventional	2/79 (2.5)
Liposomal	70/79 (88.6)
Both^a^	7/79 (8.9)
Antifungal treatment duration (d), median (percentile 25 to percentile 75)	29.0 (11.0 to 45.0)

Abbreviations: CKD/ESRD, end-stage renal disease/chronic kidney disease; HIV, human immunodeficiency virus; AIDS, acquired immunodeficiency syndrome; CT, computed tomography; ESR, erythrocyte sedimentation rate; CRP, C-reactive protein.

^a^Patients who received a specific formulation for more than 80% of total injections were considered part of that group; otherwise, they were classified in the “Both” category.

### Clinical Manifestations

The most common presenting symptoms at admission included headache in 55 patients (67.9%), facial pain in 50 patients (61.7%), facial numbness in 46 patients (56.8%), and visual disturbances in 45 patients (55.6%). On physical examination, facial swelling was the most frequent finding, observed in 57 patients (70.4%). Additional findings included ptosis in 54 patients (66.7%), periorbital swelling in 49 patients (60.5%), proptosis in 48 patients (59.3%), nasal ulceration in 35 patients (43.2%), palatal necrosis in 34 patients (42.0%), and facial paralysis in 26 patients (32.1%). Facial skin involvement was rare, occurring in only 2 patients (2.5%).

### Paranasal Sinus Involvement

A total of 54 patients (66.7%) exhibited involvement of all four paranasal sinuses, while 16 patients (19.8%) had three sinus involvements, 6 patients (7.4%) had two sinus involvements, and 5 patients (6.2%) had only one sinus affected. Bilateral sinus involvement was seen in 66 patients (81.5%), whereas unilateral involvement was present in 15 patients (18.5%). The maxillary sinus was the most frequently involved (79 patients, 97.5%), followed by the ethmoid sinus (75 patients, 92.6%), the sphenoid sinus (68 patients, 84.0%), and the frontal sinus (62 patients, 76.5%).

### Infection Extension Beyond the Paranasal Sinuses

The spread of infection beyond the paranasal sinuses was frequently observed. Eye involvement occurred in 52 patients (64.2%). Brain involvement was documented in 30 out of 66 patients who underwent neuroimaging (45.5%).

### Diagnostic Findings

Biopsy was primarily performed on the paranasal sinuses in 77 patients (95.1%), while orbit and skull base biopsies were conducted in 1 patient (1.2%) each. A combination of sinus, orbit, and skull base biopsies was performed in 2 patients (2.5%). Histopathological examination confirmed mucormycosis in 80 patients (98.8%), with only 1 patient (1.2%) having non-specific findings.

### Imaging Findings

Orbital involvement was detected in 59 patients (72.8%) on computed tomography (CT) imaging, whereas 22 patients (27.2%) had normal orbital CT results. Brain involvement on CT was observed in 26 out of 55 patients (47.3%). Of the 38 patients who underwent brain magnetic resonance imaging (MRI), 23 patients (60.5%) exhibited brain involvement. Overall, 45.5% of patients who underwent neuroimaging had evidence of brain involvement. The most common brain abnormalities included cerebral infarcts (70.3%), brain abscesses with infarcts (14.8%), cavernous sinus involvement (7.4%), brain abscesses with cavernous sinus involvement (4.7%), and isolated brain abscesses (3.7%).

### Treatment and Surgical Interventions

Corticosteroid use was recorded in 73 patients (90.1%), while remdesivir was administered to 78 patients (96.3%). Biologic medications were prescribed to 19 patients (23.5%), including tocilizumab in 14 patients (17.3%), tofacitinib in 1 patient (1.2%), and a combination of tocilizumab and tofacitinib in 4 patients (4.9%). Early antifungal treatment following hospital admission was initiated in 87.5% of patients. Antifungal therapy consisted of amphotericin B in 79 patients (97.5%), posaconazole in 15 patients (18.5%), a combination of amphotericin B and caspofungin in 14 patients (17.3%), caspofungin alone in 1 patient (1.2%), voriconazole in 1 patient (1.2%), and a combination of amphotericin B and itraconazole in 1 patient (1.2%).

Surgical debridement was performed in most cases. A total of 18 patients (22.2%) underwent a single debridement session, whereas 62 patients (76.6%) required between 2 and 11 sessions. One patient (1.2%) did not undergo surgical debridement due to rapid clinical deterioration and death before the procedure could be performed.

### Survival and Long-Term Outcomes

Among the 81 patients, 42 (51.9%) survived CAM and were discharged from the hospital. However, many of these survivors experienced persistent sequelae. Of the 23 patients (54.8%) discharged with ongoing complications, visual impairment was the most prevalent issue. A total of 22 patients (95.7%) experienced visual disturbances, including 1 patient (4.4%) with bilateral involvement and 21 patients (91.3%) with unilateral impairment. Among the latter group, 7 patients (33.3%) had reduced vision, while 14 patients (66.7%) suffered unilateral blindness.

Facial nerve involvement was identified in 12 patients (52.2%). Additionally, 2 patients (8.7%) had unilateral hemiparesis, 1 patient (4.4%) experienced hypesthesia in the lower limbs, 1 patient (4.4%) had unilateral trigeminal nerve hypesthesia, and 2 patients (8.7%) presented with extensive palatal and facial defects.

**Figure 1.**
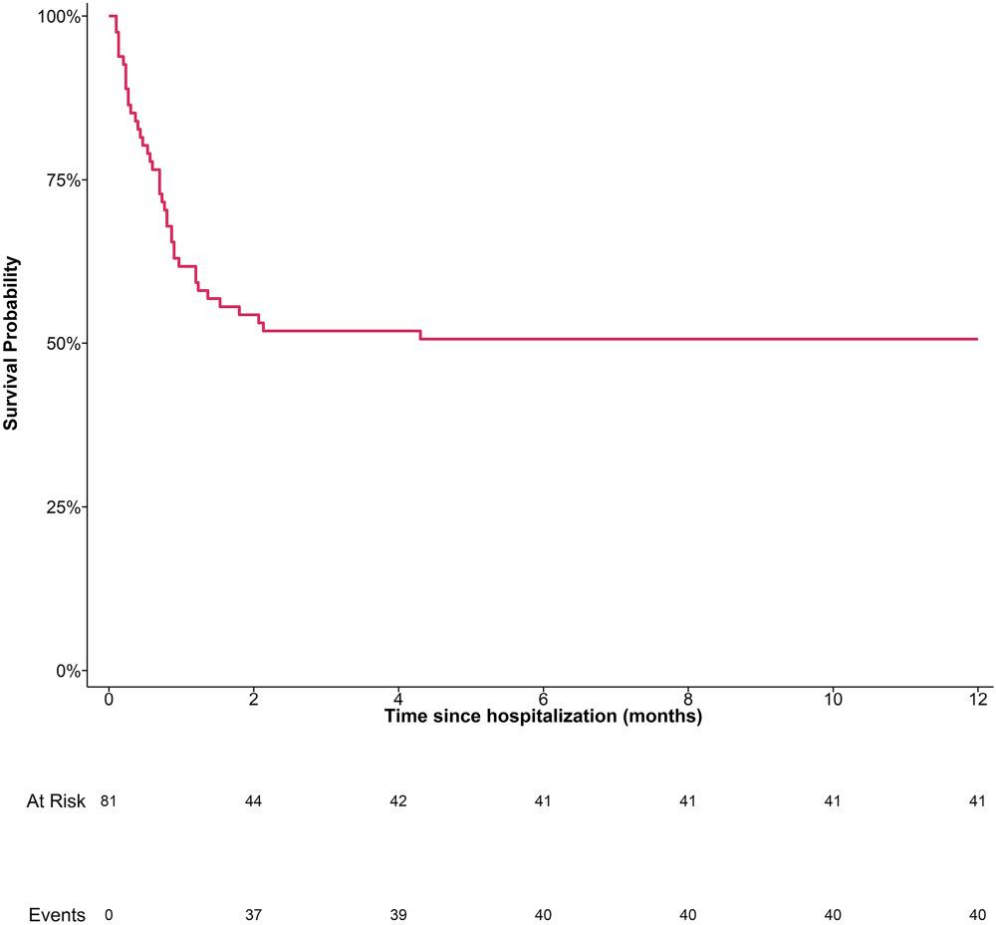
Kaplan–Meier survival curves for patients with COVID-19-associated mucormycosis. The *x*-axis indicates time (in m), while the *y*-axis shows cumulative survival probability.

Long-term survival analysis revealed a 1-month survival rate of 61.7% (95% CI: 50.2% to 71.3%), with 31 deaths recorded (Figure 1). The 3-month survival rate was 51.9% (95% CI: 40.5% to 62.1%). The survival rates at both 6 and 12 months were 50.6% (95% CI: 39.3% to 60.9%), with a total of 40 deaths.

### Predictors of One-Year Mortality

The survival regression analysis identified several factors associated with one-year mortality. In the univariable analysis, significant continuous predictors included age (*P*-value: .037), platelet count (*P*-value: .006), C-reactive protein (CRP) levels (*P*-value: .001), and treatment duration (*P*-value: < .001) (Table 2). These associations remained significant after adjusting for age.

**Table 2.** Predictors of Mortality based on an Increase in the Unit of Variable

	Univariable Analysis		Age-adjusted Analysis	
Variable	Hazard ratio (95% CI)	*P-*value	Hazard ratio (95% CI)	*P-*value
Age (y)	1.03 (1.00 to 1.06)	.037	-	-
Peripheral leukocytes (×10^3^/per µl)	1.00 (.99 to 1.01)	.383	1.01 (1.00 to 1.02)	.255
Hemoglobin (mg/dL)	1.00 (1.00 to 1.00)	.065	1.00 (1.00 to 1.00)	.057
Platelets (×10^3^/per µl)	1.00 (.99 to 1.00)	.006	1.00 (.99 to 1.00)	.007
ESR (mm/h)	1.00 (.99 to 1.01)	.513	1.00 (.99 to 1.01)	.511
CRP (mg/L)	1.01 (1.00 to 1.01)	.001	1.01 (1.00 to 1.01)	.001
Blood sugar (mg/dL)	1.00 (1.00 to 1.00)	.351	1.00 (1.00 to 1.00)	.435
Antifungal treatment duration (d)	.94 (.92 to .96)	< .001	.94 (.92 to .96)	< .001
duration between COVID-19 and initiation of CAM (d)	.98 (.95 to 1.00)	.091	.97 (.95 to 1.00)	.052

Abbreviations: CI, confidence interval; HR, hazard ratio; ESR, erythrocyte sedimentation rate; CRP, C-reactive protein; CAM, COVID-19-associated mucormycosis.

**Figure 2.**
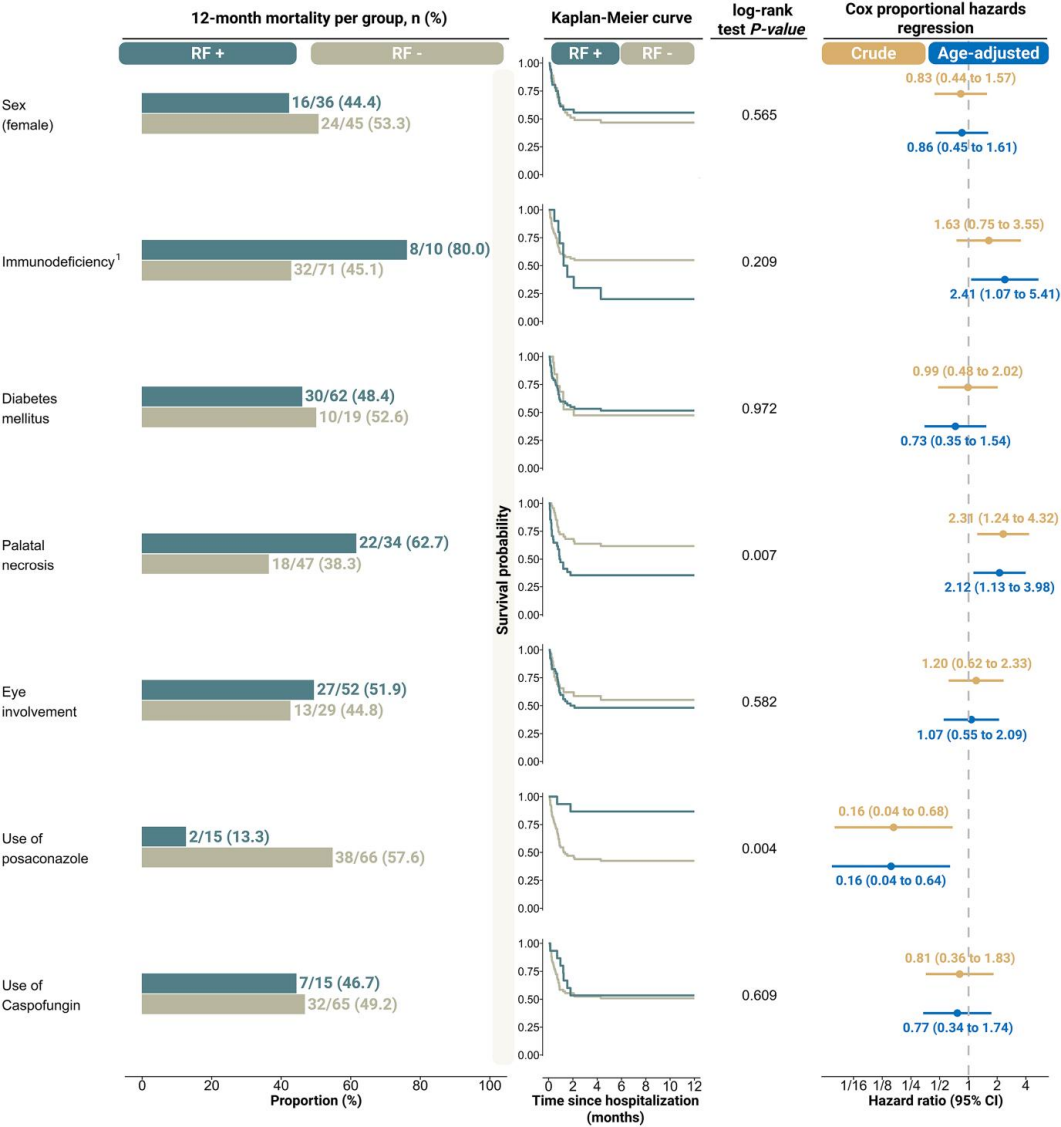
Analysis of univariable and age-adjusted models examining the relationship between different clinical and neuroimaging features and the 12-m mortality risk in patients with COVID-19-associated mucormycosis [1]. Diabetes mellitus was not considered a factor for immunodeficiency. RF, risk factor; CI, confidence interval.

Among categorical variables, palatal necrosis and the use of posaconazole were significantly correlated with mortality risk. In the unadjusted model, palatal necrosis was associated with a higher mortality risk (HR: 2.31, 95% CI: 1.24 to 4.32), whereas posaconazole use was linked to a lower mortality risk (HR: 0.16, 95% CI: 0.04 to .68) (Figure 2). These associations remained significant in the age-adjusted model (palatal necrosis: HR: 2.12, 95% CI: 1.13 to 3.98; posaconazole: HR: 0.16, 95% CI: 0.04 to .64).

**Figure 3.**
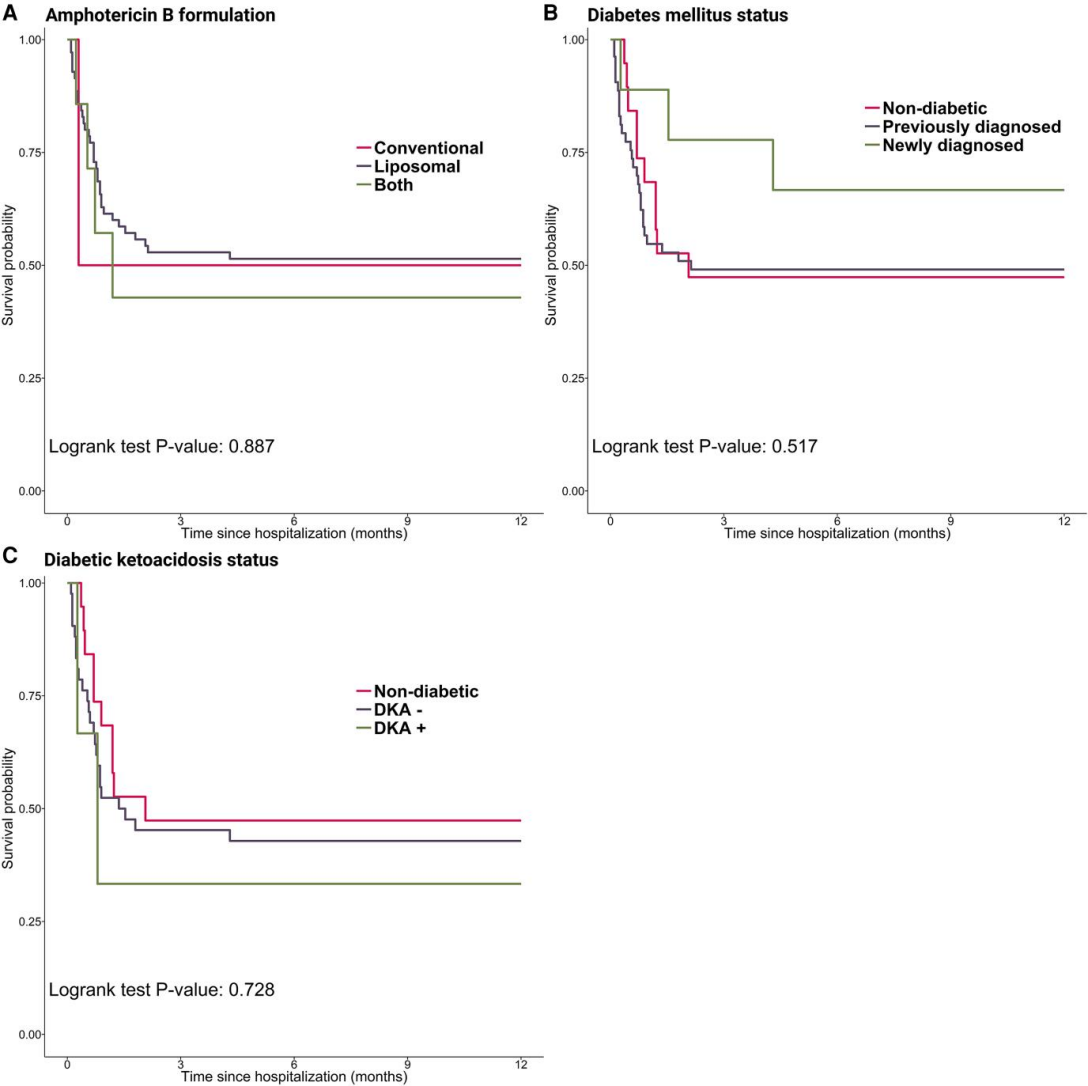
Kaplan–Meier survival curves for patients with COVID-19-associated mucormycosis based on amphotericin B formulation (*A*), diabetes status (newly diagnosed vs preexisting) (*B*), and diabetic ketoacidosis status (*C*). The *x*-axis represents time (m), and the *y*-axis represents cumulative survival probability. DKA +, presence of diabetic ketoacidosis; DKA -, absence of diabetic ketoacidosis.

The proportional hazards assumption was met for all variables except immunodeficiency. We stratified the analysis at 1 month after initial admission, which was the time point when the 2 groups (immunodeficient and immunocompetent) crossed each other. The analysis indicated that immunodeficiency was not significantly associated with mortality within the first month (HR: 0.53, 95% CI: 0.16 to 1.77), while it became a significant predictor of mortality thereafter (HR: 11.07, 95% CI: 2.94 to 41.73). Other factors, namely the formulation of amphotericin B (log-rank test *P*-value = .887), diabetes mellitus status (log-rank test *P*-value = .517), and the presence or absence of diabetic ketoacidosis (log-rank test *P*-value = .728), were not significantly associated with mortality (Figure 3*A*–*C*).

## DISCUSSION

Long-term follow-up of patients with COVID-19-associated mucormycosis (CAM) highlights the aggressive and devastating nature of this disease, with survival rates declining sharply in the early months after diagnosis and stabilizing thereafter. In this cohort, the 1-month survival was 61.7%, which dropped to 51.9% at 3 months and remained at 50.6% at both 6 and 12 months. These survival rates reflect the aggressive nature of mucormycosis, particularly when compounded by COVID-19, which exacerbates the patient's overall immunocompromised state. Importantly, survival was not synonymous with recovery; among the 42 survivors, 54.8% experienced long-term sequelae. The majority suffered persistent visual impairment (95.7%), while neurological complications were seen in 52.2% of patients, including facial nerve involvement, unilateral hemiparesis, and hypesthesia in the lower limbs or unilateral trigeminal nerve hypesthesia. Extensive palatal and facial defects were present in 8.7% of cases. These data underscore the high burden of morbidity in survivors and the need for long-term multidisciplinary management, including neurological rehabilitation, visual support, and reconstructive surgery.

Almost all cases in this series presented as rhino-orbital-cerebral mucormycosis (ROCM), which remains the predominant form of CAM. This pattern is consistent with previous reports describing ROCM as the most severe and life-threatening presentation of mucormycosis, often originating in the paranasal sinuses before spreading to the orbit and central nervous system [10]. Survival rates for COVID-19-associated ROCM vary widely between studies, ranging from 20% to 80% [10–12]. In the present study, the 6-month survival rate of 50.6% translates to a mortality rate of 49.4%. A systematic review reported a 37.3% mortality rate for COVID-19-associated ROCM despite aggressive antifungal therapy and surgical debridement [13]. Reports from Iran have shown highly variable mortality outcomes ranging from 11.8% to 59.8% [14–16]. Such heterogeneity may be explained by differences in early recognition, availability of imaging, timeliness of antifungal therapy, and extent of surgical intervention. Early and aggressive management is crucial in improving outcomes for patients with ROCM and highlights the need for heightened awareness and swift action in managing this devastating infection, particularly in the context of COVID-19.

Our Cox regression analysis identified key predictors of 12-month mortality in CAM. Elevated CRP levels, palatal necrosis, immunodeficiency, and shorter antifungal therapy during hospitalization were associated with increased mortality, while use of posaconazole improved survival. In multivariable analysis, CRP elevation, palatal necrosis, and limited antifungal duration remained independent risk factors, whereas posaconazole use retained its protective association. The survival benefit of posaconazole may stem from its broad antifungal activity and feasibility as an oral step-down therapy, enabling longer treatment courses beyond hospital discharge. Although our study did not include data on post-discharge antifungal therapy, patients receiving posaconazole may have undergone longer total treatment durations, which could have contributed to better outcomes. However, its cost and limited availability in resource-constrained settings restricted use, and selection bias cannot be excluded since posaconazole was more likely prescribed to stable patients who could tolerate oral medications. Nonetheless, even after adjusting for confounders, its protective role persisted.

The impact of immunodeficiency demonstrated an interesting temporal pattern. While it was not significantly associated with mortality during the first month (HR 0.53), it emerged as a strong predictor thereafter (HR 11.07). This suggests that early deaths are largely attributable to fulminant fungal invasion, delayed diagnosis, or suboptimal initial therapy—factors affecting both immunocompetent and immunocompromised patients. Over the longer term, however, impaired immune status becomes more critical, leading to persistent infection, delayed recovery, and secondary complications [3]. This finding highlights the need for both aggressive early therapy and prolonged antifungal treatment in immunodeficient individuals. Notably, longer antifungal treatment durations were associated with reduced mortality risk. This finding underscores the importance of prolonged antifungal therapy in improving survival outcomes in CAM patients.

Clinical manifestations of ROCM in this cohort closely resembled prior descriptions. The most frequent symptoms were facial swelling (70%), headache (68%), and facial pain (62%), consistent with other reports that emphasized ocular pain, ophthalmoplegia, proptosis, necrosis, and sinusitis-like features [12, 17–19]. Given the wide range of presentations, clinicians should maintain a high index of suspicion for mucormycosis in COVID-19 patients, particularly those with diabetes or a history of corticosteroid or immunosuppressant use. Unusual signs—such as severe dental pain or sudden tooth loosening—also warrant evaluation. Notably, one patient in our study extracted three of their own teeth due to intense pain, leading to partial palatal loss, emphasizing the importance of recognizing even atypical presentations of mucormycosis.

Diagnosis of mucormycosis remains challenging, especially when relying on cultures, which have low sensitivity. A systematic review demonstrated culture positivity in only 37% of 476 CAM cases [20]. Histopathological confirmation, therefore, remains the diagnostic gold standard. In the present series, mucormycosis was confirmed histologically in 98.8% of cases, predominantly from initial sinus biopsies (95%). We did not report specific culture positivity rates, as tissue fungal cultures are not routinely performed in our centers due to limited resources and infrastructure. These results align with Iranian data reporting biopsy confirmation in 100% of cases [21]. In a separate study of 200 biopsy-confirmed CAM patients, common histological findings included necrosis, granuloma, vascular invasion, and perineural invasion, with fungal cultures positive in 86%. Importantly, histological features have been linked to mortality, underscoring their prognostic as well as diagnostic significance [22].

Imaging is indispensable in assessing disease extent and guiding treatment. CT in this cohort revealed paranasal sinus involvement in 97.5% of patients, with all four sinuses affected in 67% and bilateral disease in 81.5%. The maxillary sinus was most frequently involved (97.5%), followed by the ethmoid (92.6%), sphenoid (84.0%), and frontal sinuses (76.5%). Orbital involvement was present in 72.8%. In a subset of 38 patients undergoing brain MRI, 60.5% had brain involvement.

Our findings are consistent with prior studies reporting similar patterns of sinus and orbital involvement in CAM [12, 22–24]. The ethmoid and maxillary sinuses are frequently affected, and ethmoid involvement is particularly significant due to its anatomical proximity to the orbit, increasing the risk of complications such as orbital cellulitis, proptosis, and extraocular muscle involvement [25]. These data underscore the importance of imaging not only in diagnosis but also in assessing disease progression and guiding clinical decisions. Given the high mortality associated with orbital and intracranial spread, prompt and comprehensive imaging evaluation is critical for effective management of mucormycosis in at-risk populations.

CNS (central nervous system) involvement in mucormycosis typically results from contiguous spread from the paranasal sinuses and orbits, while hematogenous dissemination accounts for approximately 30% of cases [26]. Fungal invasion can lead to severe cerebrovascular complications, including cerebral infarcts, intracranial hemorrhages, arterial occlusion, mycotic aneurysms, and brain abscesses [27]. The incidence of CNS mucormycosis varies by population, being most common among intravenous drug users, followed by patients with diabetes, malignancies, organ transplants, and trauma [28]. In our study, brain CT scans performed on 55 of 81 patients revealed CNS involvement in 47.3%, while contrast-enhanced brain MRI in 38 patients showed involvement in 60.5%. Overall, 45.5% of patients who underwent neuroimaging had CNS manifestations, including cerebral infarcts (70.3%), brain abscesses with infarcts (14.8%), cavernous sinus involvement (7.4%), brain abscesses with cavernous sinus involvement (4.7%), and isolated brain abscesses (3.7%).

Compared to previous studies, our findings indicate a higher rate of brain abscess formation. One MRI-based study of 63 CAM patients reported intracranial involvement in only 11.2%, including cavernous sinus involvement (1.6%), cavernous ectasia (1.6%), focal cerebritis (1.6%), and stroke (4.8%) [29]. A single-center study in Iran involving 94 CAM cases identified cavernous sinus thrombosis, cerebritis, brain abscess, and cerebral infarction as the most common CNS complications [30]. Ethmoid sinus involvement increases the risk of cavernous sinus thrombosis, while sphenoid infection may extend to the cavernous sinus and carotid artery, potentially reaching the frontoparietal region [31]. Less frequent complications include sagittal sinus thrombosis, subdural or epidural abscesses, and rarely, meningitis with obstructive hydrocephalus [28, 32]. These underscores the importance of early and comprehensive imaging to guide appropriate therapeutic interventions in mucormycosis cases.

Underlying risk factors in this cohort paralleled global experience. Diabetes mellitus was the most prevalent comorbidity, affecting 77% of patients, reaffirming its role as the principal predisposing condition in CAM. Reports from India, Iran, and Mexico consistently identify uncontrolled diabetes and hyperglycemia as the leading contributors [25]. Corticosteroid use was reported in over 90% of this cohort, consistent with rates of 76%–85% in other series [10, 21]. One-quarter of patients also received immunosuppressive therapies such as tocilizumab and tofacitinib. A systematic review from India reported corticosteroid exposure in 76.3% and tocilizumab use in 14.1% [23]. A large case–control study of 267 mucormycosis patients and 256 controls demonstrated a 3.2-fold increased risk of infection associated with corticosteroids, independent of dosage [33]. The combination of corticosteroid-induced hyperglycemia, metabolic acidosis, and impaired immune function creates an ideal environment for fungal proliferation [14]. These observations stress the importance of cautious steroid prescribing and strict glucose control in COVID-19 patients.

Management of CAM requires a rapid, aggressive, and multidisciplinary approach. Correcting underlying risk factors, withdrawing immunosuppressive drugs, initiating antifungal therapy without delay, and performing surgical debridement are all central to successful outcomes [17, 25]. Surgical intervention, often repeated, is particularly important. Radical surgery has been shown to achieve local control in 90% of cases, compared with 41.6% for limited procedures [27], and can reduce mortality by up to 13% even in advanced cases [34]. In this cohort, early antifungal therapy was initiated in 87.5% of patients. Amphotericin B was the mainstay, administered to 97.5% of patients, sometimes in combination with agents such as posaconazole or caspofungin. Amphotericin B remains the most effective agent against mucorales in vitro and in vivo [35], though clinical outcomes in humans remain heterogeneous. Combination therapies, particularly amphotericin B with caspofungin, have shown encouraging results in rhino-orbital-cerebral disease [28], but given intrinsic resistance patterns, azoles and echinocandins are not reliable as monotherapies [27]. Timely initiation of antifungal treatment is critical, as delays beyond six days have been associated with a near doubling of mortality [36]. Together, these data reinforce the necessity of early, aggressive, and comprehensive therapy in CAM.

This study has several limitations that must be acknowledged. First, as a retrospective cohort study, it is subject to inherent biases, including selection bias and information bias, which may impact the accuracy of the data collected. Second, the sample size, although significant, may not be large enough to fully capture the diversity of clinical presentations and outcomes across different populations. Third, our study did not include a control group of COVID-19 patients without mucormycosis, which limits the ability to directly compare the risk of mortality and other outcomes between these groups. Fourth, the study was conducted in a referral center in Mashhad, Iran, which may limit the generalizability of our findings to other regions with different healthcare infrastructures and access to antifungal treatments. Additionally, some clinical and biochemical data—such as the exact duration and dose of corticosteroid use and the degree of glycemic control during hospitalization—were not available for all patients, which may have limited our ability to fully assess the impact of these factors on survival. Lastly, despite the identification of elevated CRP as a predictor of mortality, the underlying mechanisms by which inflammatory markers influence outcomes in CAM require further exploration in prospective studies to refine our understanding of this association.

## CONCLUSIONS

Our study contributes to the growing body of evidence on the clinical and epidemiological characteristics of CAM, emphasizing the critical need for early diagnosis, identification of risk factors, and prompt, prolonged treatment to improve patient outcomes. Elevated CRP, reduced antifungal treatment duration, and palatal necrosis were identified as predictors of higher mortality in CAM. In addition, the use of posaconazole was associated with a protective effect against mortality. Future studies should focus on refining risk stratification and optimizing therapeutic protocols to mitigate the high mortality associated with this devastating disease.
